# A Conserved LIR Motif in Connexins Mediates Ubiquitin-Independent Binding to LC3/GABARAP Proteins

**DOI:** 10.3390/cells9040902

**Published:** 2020-04-07

**Authors:** Steve Catarino, Teresa M Ribeiro-Rodrigues, Rita Sá Ferreira, José Ramalho, Christine Abert, Sascha Martens, Henrique Girão

**Affiliations:** 1 Coimbra Institute for Clinical and Biomedical Research (iCBR), Faculty of Medicine, University of Coimbra, 3000-548 Coimbra, Portugal; scatarino@fmed.uc.pt (S.C.); teresamrrodrigues@gmail.com (T.M.R.-R.); rita_saferreira@hotmail.com (R.S.F.); 2 Center for Innovative Biomedicine and Biotechnology (CIBB), University of Coimbra, 3000-548 Coimbra, Portugal; 3 Clinical Academic Centre of Coimbra (CACC), University of Coimbra, 3000-548 Coimbra, Portugal; 4 CEDOC, Chronic Diseases Research Centre, NOVA Medical School|Faculdade de Ciências Médicas, Universidade NOVA de Lisboa, 1150-082 Lisboa, Portugal; jose.ramalho@nms.unl.pt; 5 Department of Biochemistry and Cell Biology, Max Perutz Labs, University of Vienna, 1030 Vienna, Austria; chr.abert@gmail.com (C.A.); sascha.martens@univie.ac.at (S.M.)

**Keywords:** autophagy, Cx43, GABARAP, gap junction, MAPLC3

## Abstract

Gap junctions (GJ) are specialized cell-cell contacts formed by connexins (Cxs), which provide direct communication between adjacent cells. Cx43 ubiquitination has been suggested to induce the internalization of GJs, as well as the recruitment of the autophagy receptor p62 to mediate binding to LC3B and degradation by macroautophagy. In this report, we describe a functional LC3 interacting region (LIR), present in the amino terminal of most Cx protein family members, which can mediate the autophagy degradation of Cx43 without the need of ubiquitin. Mutation of the LIR motif on Cx37, Cx43, Cx46 and Cx50 impairs interaction with LC3B and GABARAP without compromising protein ubiquitination. Through in vitro protein-protein interaction assays, we demonstrate that this LIR motif is required for the binding of Cx43 to LC3B and GABARAP. Overall, our findings describe an alternative mechanism whereby Cxs interact with LC3/GABARAP proteins, envisioning a new model for the autophagy degradation of connexins.

## 1. Introduction

Connexins (Cx) are a family of proteins that directly connect the cytoplasm of adjacent cells through the formation of gap junction (GJ) channels. In humans, twenty members form the connexin family, while at least seventeen members have been reported in rats. Based on sequence homology, connexin genes can be grouped into four classes, α, β, γ and δ [1]. Each connexin consists of four transmembrane regions, two extracellular loops and one intracellular loop, with both the amino and carboxyl terminals facing the cytosol. After synthesis, connexin proteins oligomerize into hexameric structures termed hemichannels and are transported to the plasma membrane, where they may dock to hemichannels from adjacent cells to form a functional GJ channel. The carboxyl terminal is where most differences between connexins are found, and a large part of the protein interactions and post-translational modifications of connexins are thought to occur in this region [2]. Although traditionally associated with establishing direct communication between adjacent cells, the localization of connexins at the plasma membrane as undocked connexin hemichannels as well as its presence in mitochondria, nucleus and extracellular vesicles, suggest that connexins play other non-canonical biological roles [3]. Given all of the above, fine-tuned regulatory mechanisms are required to modulate connexin levels, function and localization.

Although GJ intercellular communication can be modulated through the gating of the channel pore, mechanisms that regulate the number of connexin-containing channels at the plasma membrane have also been implicated in the regulation of intercellular communication. Given the short half-life of Cx43, mechanisms of protein degradation play an important role in the regulation of Cx43 levels and intercellular communication. We and others have shown that ubiquitination of Cx43, modulated by E3 ligases, including neural precursor cell expressed developmentally down-regulated protein 4 (Nedd4), and deubiquitinating enzymes, such as associated molecule with the SH3 domain of STAM (AMSH), dictates the final fate of the protein [4,5,6,7]. At the plasma membrane, Cx43 is modified by Lysine 63-linked polyubiquitin chains, which are recognized by the endocytic adaptor epidermal growth factor receptor substrate 15 (Eps15) to trigger the internalization of the protein [7]. Furthermore, Cx43 sorting from early endosomes to the lysosome is also reliant on ubiquitination to mediate the interaction with the endosomal sorting complex required for transport (ESCRT) components hepatocyte growth factor-regulated tyrosine kinase substrate (Hrs) and tumour susceptibility gene 101 (Tsg101) [8,9]. The role of Cx43 ubiquitination in signalling lysosomal GJ degradation is important not only in the endocytic pathway but also in the autophagy process. Indeed, GJ degradation by macroautophagy is also dependent, at least partially, on prior ubiquitination of Cx43 [10,11], a process that requires not only the ubiquitin-binding domain containing autophagy receptor p62, but also, and surprisingly, the endocytic adaptor Eps15 [11]. However, preventing the ubiquitination of Cx43 did not fully abrogate its autophagic degradation, suggesting that alternative mechanisms for targeting Cx43 to autophagosomes exist.

Autophagy is one of the major catabolic pathways in the cell, in which substrates are delivered to the lysosome for degradation. In macroautophagy, a double membrane structure termed the phagophore, grows around the target substrate, eventually closing to form an autophagosome, which subsequently fuses with the lysosome. During phagophore formation, the ubiquitin-like protein microtubule-associated protein 1 light chain 3 (MAPLC3) is conjugated to phosphatydilethanolamine (PE) present on the nascent phagophore, through a mechanism closely resembling ubiquitin conjugation. The E1-like protein autophagy-related protein 7 (ATG7) activates LC3, to allow its transfer to the E2-like protein ATG3. In the final step of the conjugation process, the E3-like complex ATG12-ATG5-ATG16L1 facilitates the transfer of LC3 to PE [12]. The LC3/GABARAP (γ-aminobutyric acid receptor–associated protein) family is comprised of 6 members in humans: LC3A, LC3B, LC3C, GABARAP, GABARAPL1 and GABARAPL2. Although macroautophagy was initially thought of as an unselective process for the bulk degradation of cytosolic content, it is now understood that this process can be highly selective. Substrates to be degraded are labelled with ubiquitin chains, which, in turn, are recognized by ubiquitin-binding domains on autophagy receptors such as p62, neighbour of BRCA1 gene 1 (NBR1), NDP52 and Optineurin. These receptors bridge the substrate to the nascent phagophore by binding to LC3/GABARAP family proteins present in the inner membrane through LC3-interacting region (LIR) motifs. The core consensus sequence of the LIR motif consists of [W/F/Y]-X1-X2-[L/I/V] [12,13]. More recently, a GABARAP interacting motif (GIM) has been described, consisting of the consensus sequence [W/F]-[V/I]-X2-V [14]. Although the ubiquitin-dependent binding of Cx43 to LC3 was previously shown to be mediated by p62 [11], the existence of a putative LIR motif in the amino terminal as well as the fact that hampering Cx43 ubiquitination and p62 silencing does not completely abolish the interaction of Cx43 with LC3, prompted us to investigate whether Cx43 can directly bind to LC3 through a LIR motif, without the need of ubiquitin.

In this study we identify a functional LIR domain in several members of the connexin family, which mediates binding to both LC3B and GABARAP proteins. We also show that connexin ubiquitination alone is insufficient to promote binding to LC3 proteins. Mutation of the LIR motif of Cx43 also rendered the protein resistant to nutrient deprivation-induced degradation. Altogether, the results presented herein support a model in which connexin proteins are targeted for autophagosomal degradation through direct binding to LC3/GABARAP family proteins present on the nascent phagophore membrane.

## 2. Materials and Methods

### 2.1. Antibodies and Reagents

Goat polyclonal antibodies against Cx43 (AB1600), GST (AB9919-200), V5 (AB0096-500) and calnexin (AB0041-500) were obtained from SICGEN (Cantanhede, Portugal). Rabbit polyclonal antibodies against Cx43 (H-150) and mouse monoclonal antibodies against p62 (sc-28359) were obtained from Santa Cruz Biotechnology (Heidelberg, Germany). Rabbit polyclonal antibodies against GABARAP (ab109364) and Nedd4 (ab14592) were obtained from Abcam (Cambridge, UK). Rabbit polyclonal antibodies against LC3B (PA1-16930) and mouse monoclonal antibodies against V5 (46-0705) were obtained from Invitrogen (Paisley, UK). Mouse monoclonal antibodies against ubiquitin (P4D1) were obtained from Covance (San Diego, CA, USA). Mouse monoclonal antibodies against EEA1 (610457) were obtained from BD Transduction Laboratories (San Jose, CA, USA). Rabbit polyclonal antibodies against ATG7 (A2856), Phorbol 12-myristate 13-acetate (PMA), 4′,6-Diamidino-2-phenylindole dihydrochloride (DAPI) and cycloheximide (CHX) were obtained from Sigma (Saint Louis, MO, USA). Bafilomycin A1 was obtained from Bioaustralis (Smithfield, Australia).

### 2.2. Cell Culture and Transfections

HEK293A cells were cultured in Dulbecco’s modified Eagle’s medium (DMEM) supplemented with 10% fetal bovine serum and antibiotics (100 U/mL penicillin, 100 g/mL streptomycin and 8 μg/mL blasticidin) and maintained at 37 °C under 5% CO_2_. Transient transfection of cells was performed with Lipofectamine 2000 (Grand Island, NY, Invitrogen), according to the manufacturer’s recommendations.

### 2.3. Plasmid Constructions

Rat Cx43 cDNA was cloned into a modified pENTR GFP C2 vector [15]. Site-directed mutagenesis was performed to generate the Cx43^W4A+L7A^, Cx43^Y265A^, Cx43^Y286A^ and Cx43^Y265,286A^ mutants from Cx43 cDNA. Plasmids expressing V5-Cx43^WT^ and V5-Cx43^W4A+L7A^ were generated by cloning the appropriate cDNA into a pENTR V5 vector. Rat Cx37 and Cx40 cDNA were amplified from rat aorta tissue using RT-PCR and cloned into a pENTR V5 vector. Rat Cx46 and Cx50 cDNA were amplified from rat lens using RT-PCR and cloned into a pENTR V5 vector. Site-directed mutagenesis was performed to generate the W4A+L7A mutants from Cx37, Cx40, Cx46 and Cx50. Plasmids expressing GST-Cx43^WT^_NT and GST-Cx43^W4A+L7A^_NT were generated by subcloning the first 22 amino acids of Cx43^WT^ and Cx43^W4A+L7A^ into a pGEX4T1 vector. All constructs were verified by DNA sequencing. GFP-LC3B and GFP-GABARAP were obtained by insertion of human LC3B and GABARAP cDNAs into pEGFP-C1. Fusion proteins were subsequently cloned into pETDuet-1 for bacterial expression. The last five amino acids of the LC3B coding sequence and the last amino acid of GABARAP were deleted to mimic ATG4 cleavage. A 6xHis-tag was added C-terminally to purify the proteins [16]. Plasmids expressing GFP-LC3B were kindly provided by Dr. Tamotsu Yoshimori (National Institute for Basic Biology, Okazaki, Japan).

### 2.4. Viral shRNA Infection

HEK293A cells were incubated with the lentiviral vectors and 8 µg/mL of polybrene for 20 min at room temperature. After 30 min centrifugation at 800× *g* and 32 °C, cells were plated and monitored for the expression of GFP. Lentiviral vectors containing shRNA targeting ATG7 and the control empty vector were kindly provided by Dr. A.M. Cuervo (Albert Einstein College of Medicine, Yeshiva University, New York, USA).

### 2.5. siRNA-Mediated Knockdown

siRNA targeting p62 (s16960 or s16961) and a non-targeting control sequence were obtained from Ambion. Cells were transfected with 20 nM siRNA using Lipofectamine 2000 (Grand Island, NY, Invitrogen) according to manufacturer’s recommendations. p62 knockdown was achieved after 24 h of transfection.

### 2.6. Immunoprecipitation and Western Blotting

Cells were rinsed with phosphate buffered saline (PBS) at 4 °C, resuspended in lysis buffer (190 mM NaCl, 50 mM Tris-HCl, 6 mM EDTA, 1% Triton X-100, pH 8.3) supplemented with protease inhibitor cocktail (Roche), 2 mM PMSF, 10 mM iodoacetamide, and incubated on ice for 10 min. The samples were then centrifuged at 10,000× *g* for 10 min and the supernatants used for immunoprecipitation. Briefly, protein G was incubated with goat polyclonal antibodies directed against Cx43 or V5. Incubations proceeded for 1 h at 4 °C, followed by incubation with supernatants for 3 h at 4 °C. The samples were then centrifuged and the protein G-sepharose sediments washed 3 times in an appropriate washing buffer (500 mM NaCl, 50 mM Tris-HCl, 6 mM EDTA, 1% Triton X-100, pH 8.3), resuspended in Laemmli buffer and denatured at 70 °C for 10 min.

For Western blot analysis of the immunoprecipitated proteins, samples were separated using SDS-PAGE, transferred to a nitrocellulose membrane and probed with appropriate antibodies. Inputs represent about 10% of the total amount of protein in the lysates before immunoprecipitation. Immunoprecipitation controls were performed by pooling the lysates of two samples transfected and/or treated in the same conditions, separating them in two fractions and then proceeding with the immunoprecipitation without adding antibody to one of the samples (No Ab). The corresponding pooled lysate appears in the Western blot panels to the right of the No Ab samples.

### 2.7. Bacterial Protein Expression and Purification

GST-Cx43^WT^_NT and GST-Cx43^W4A+L7A^_NT proteins were expressed in Escherichia coli BL21-CodonPlus (DE3)-RILP Cells (Agilent Technologies, Santa Clara, CA, USA). Bacteria were grown in Luria broth (LB) medium until OD600 ≈ 0.8–1, induced with 0.1 mM isopropylthiogalactoside (IPTG) and grown at 37 °C for 4 h. GST constructs were isolated from harvested cells using Glutathione Sepharose 4B (GE Healthcare, Buckinghamshire, UK) according to manufacturer’s recommendations. GFP-LC3B and GFP-GABARAP proteins were expressed in Escherichia coli Rosetta (DE3) pLysS cells. Cells were induced at an OD600 of 0.5 for 16 h at 18 °C with 0.1 mM IPTG. Harvested cells were resuspended in lysis buffer 50 mM 4-(2-hydroxyethyl)-1-piperazineethanesulfonic acid (HEPES) at pH 7.5, 500 mM NaCl, 10 mM imidazole, 2 mM MgCl_2_, 2 mM β-mercaptoethanol, complete protease inhibitor (Roche, Basel, Switzerland) and DNase I and lysed using a freeze–thaw cycle followed by brief 30 s sonication. Lysates were cleared using ultracentrifugation at 140,000 g for 30 min at 4 °C (Beckman, Brea, CA, USA, Ti45 rotor). Supernatants were applied to Ni-NTA columns (GE Healthcare, Buckinghamshire, UK), and constructs were eluted via a stepwise imidazole gradient (50, 75, 100, 150, 200, and 300 mM) [16].

### 2.8. GFP-Trap Pull-Down Assay

Five microlitres of GFP-Trap beads slurry (ChromoTek, Munich, Germany) were mixed with a 5 μM solution of GFP-fused bait proteins (GFP-LC3B or GFP-GABARAP) and incubated on a rotating wheel at 4 °C for 1 h. Subsequently the beads were washed twice with 150 mM NaCl, 50 mM Tris at pH 7.4, and incubated with 20 μg of prey solution (GST-Cx43^WT^_NT or GST-Cx43^W4A+L7A^_NT). Precipitates were analysed using Western blot using goat polyclonal antibodies against GST.

### 2.9. Microscopy-Based Protein-Protein Interaction Assay

Five microlitres of glutathione Sepharose 4B beads slurry (GE Healthcare, Buckinghamshire, UK) were mixed with 20 µg of GST-fused bait proteins (GST-Cx43^WT^_NT or GST-Cx43^W4A+L7A^_NT) and incubated on a rotating wheel at 4 °C for at least 30 min. Subsequently the beads were washed twice with 150 mM NaCl, 50 mM Tris at pH 7.4 and resuspended in 6 µL of the same buffer. Of a 5 µM prey solution (GFP-LC3B or GFP-GABARAP), 10 µL was plated into the well of a 384-well glass-bottom microplate (Greiner Bio-One, Frickenhausen, Germany), after which 10% of the previous beads solution was added. Samples were then imaged on a confocal microscope. To quantify the protein recruitment to beads the maximum brightness along a straight line drawn through a single bead was taken (maximal fluorescence). Next, the average brightness of an empty portion of each picture was measured (background fluorescence) and subtracted from the maximal fluorescence for each bead [16].

### 2.10. Triton X-100 Fractionation Assay

The detergent solubility assay with 1% Triton X-100 was performed essentially as described previously by others [17]. Cells were resuspended in lysis buffer (190 mM NaCl, 50 mM Tris-HCl, 6 mM EDTA, 1% Triton X-100, pH 8.3) supplemented with protease inhibitor cocktail (Roche, Basel, Switzerland), 2 mM PMSF and 10 mM iodoacetamide. Samples were then ultracentrifuged at 100,000 g for 50 min and the supernatant recovered (Triton X-100 soluble fraction). The detergent-insoluble pellets were resuspended in lysis buffer supplemented with 0.1% SDS (Triton X-100 insoluble fraction) and sonicated. Laemmli buffer was added to 10% of each Triton X-100 soluble and insoluble fractions and denatured at 70 °C for 10 min before SDS-PAGE analysis. The remainder of each sample was used for immunoprecipitation.

### 2.11. Immunofluorescence

HEK293A cells grown on glass coverslips were fixed with 4% paraformaldehyde in PBS. The samples were then washed with PBS, permeabilised with 0.2% v/v Triton X-100 in PBS and blocked with 2% w/v BSA in PBS for 20 min prior to incubation with primary antibodies overnight at 4 °C. The samples were then washed three times with PBS before incubation with the secondary antibody for 1 h at room temperature. The specimens were rinsed in PBS and mounted with MOWIOL 4-88 Reagent (Calbiochem, San Diego, CA, USA). Nuclei were stained with DAPI. For controls primary antibodies were omitted. Imaging was performed on a laser-scanning confocal (Zeiss LSM 710, Carl Zeiss, Oberkochen, Germany) with Plan-Apochromat 63X/1.4 oil objective (Carl Zeiss, Oberkochen, Germany). Colocalization of Cx43 and GFP-LC3B or EEA1 was quantitated using Pearson’s correlation coefficient (PCC) using the Coloc 2 plugin of ImageJ software (http://imagej.net/Coloc_2). Cells were selected as regions of interest (ROIs) using the freehand selection tool before running the plugin. Pearson’s R values (no threshold) reported were plotted in a graph.

### 2.12. Statistical Analysis

Data were displayed with means on a scatter dot plot. For data with non-Gaussian distribution, statistical significance was determined using a non-parametric Kruskall-Wallis test followed by a Dunn’s multiple comparison test, non-parametric Mann-Whitney test or multiple Student’s t-test with Holm-Sidak’s correction. Data was analysed and graphs were assembled with GraphPad Prism 6 for Windows, version 6.01 (GraphPad Software, Inc., San Diego, CA, USA). Results were considered significantly different for *p* < 0.05. The tests used in each experiment are indicated in the figure legends. Graphical data depict individual experiments except when noted in the figure legend.

## 3. Results

### 3.1. The Connexin Protein Family Contains a Conserved LIR Motif in Its Amino Termini

**Figure 1:**
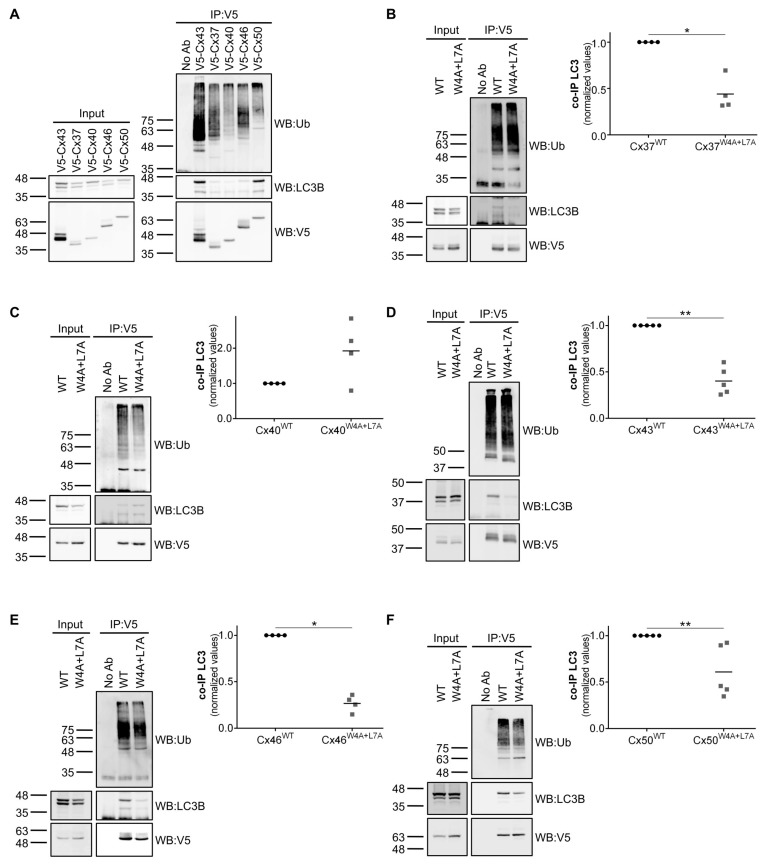
Connexin family members interact with LC3B through a LC3-interacting region (LIR) motif on their amino terminal. HEK293A cells were co-transfected with several different V5-connexin constructs and GFP-LC3B. Cell lysates were then subjected to immunoprecipitation with goat polyclonal antibodies against V5. The precipitates were analysed using Western blot using mouse monoclonal antibodies against V5 and ubiquitin, or rabbit polyclonal antibodies against LC3B (**A**). HEK293A cells were co-transfected with GFP-LC3B and V5 constructs of either wild type or W4A+L7A mutants of Cx37 (**B**), Cx40 (**C**), Cx43 (**D**), Cx46 (**E**) and Cx50 (**F**). Cell lysates were then subjected to immunoprecipitation with goat polyclonal antibodies against V5. The precipitates were analysed using Western blot using mouse monoclonal antibodies against V5 and ubiquitin, or rabbit polyclonal antibodies against LC3B. The level of co-immunoprecipitated LC3B with each connexin was quantified, normalized to the immunoprecipitated connexin and plotted. *p < 0.05, **p < 0.01, ns: non-significant using the Mann‒Whitney test.

In silico analysis of the amino acid sequence of rat Cx43 through the iLIR webtool [18] revealed a potential LIR motif in the amino terminal of the protein with core residues corresponding to tryptophan 4 (W4) and leucine 7 (L7). Further analysis of the amino acid sequence of the human [1] and rat connexin families (Figure S1) evidenced that these residues were strongly conserved within the family, being present in 17 of the 20 human connexins and 15 of the 17 rat connexins. Given these observations we proceeded to explore the interaction between several connexins of the alpha subfamily with LC3B. For this purpose we transiently transfected human embryonic kidney 293A (HEK293A) cells (which express low levels of endogenous Cx43) with a vector encoding a green fluorescent protein (GFP) tagged LC3B chimera (to overcome the low level expression of LC3B in this cell line), in addition to several connexins tagged with V5. Lysates were then subjected to immunoprecipitation against the V5 tag, after which we assessed the levels of co-immunoprecipitated GFP-LC3B. As depicted in Figure 1A, all connexins tested (Cx37, Cx40, Cx43, Cx46 and Cx50) interact with LC3B. It has been shown that the nature of the amino acids surrounding the core LIR motif, namely where hydrophobicity and charge is concerned, affects binding to LC3 [13]. Therefore, it is conceivable that variations in the levels of interaction with LC3 among the different connexins tested reflect differences in the amino acids surrounding the LIR motif on each connexin. Importantly, we also show that all of these connexins are modified with ubiquitin, which, to our knowledge, has never been reported for Cx37, Cx46 and Cx50 [4,19]. Having established that connexin binds to LC3B, we then investigated whether the putative LIR motif found in the amino terminal of these proteins was important to mediate this interaction. For this purpose we used site-directed mutagenesis to mutate the core LIR motif residues tryptophan 4 and leucine 7 to alanine, to generate the corresponding Cx^W4A+L7A^ mutants. As depicted in Figure 1B–F, mutation of the LIR motif in all connexins tested, with the exception of Cx40, led to a substantial decrease in the amount of LC3B that is co-immunoprecipitated. Given our previous observation that Cx43 ubiquitination induces its interaction with LC3B [11], there was the possibility that mutation of the LIR motif was reducing the interaction with LC3B due to a defect in ubiquitination. However, the W4A+L7A mutation had no significant effect on the ubiquitination levels of all connexins tested (Figure 1B–F).

### 3.2. Cx43 Amino Terminal Peptides Interact with LC3B and GABARAP In Vitro

**Figure 2:**
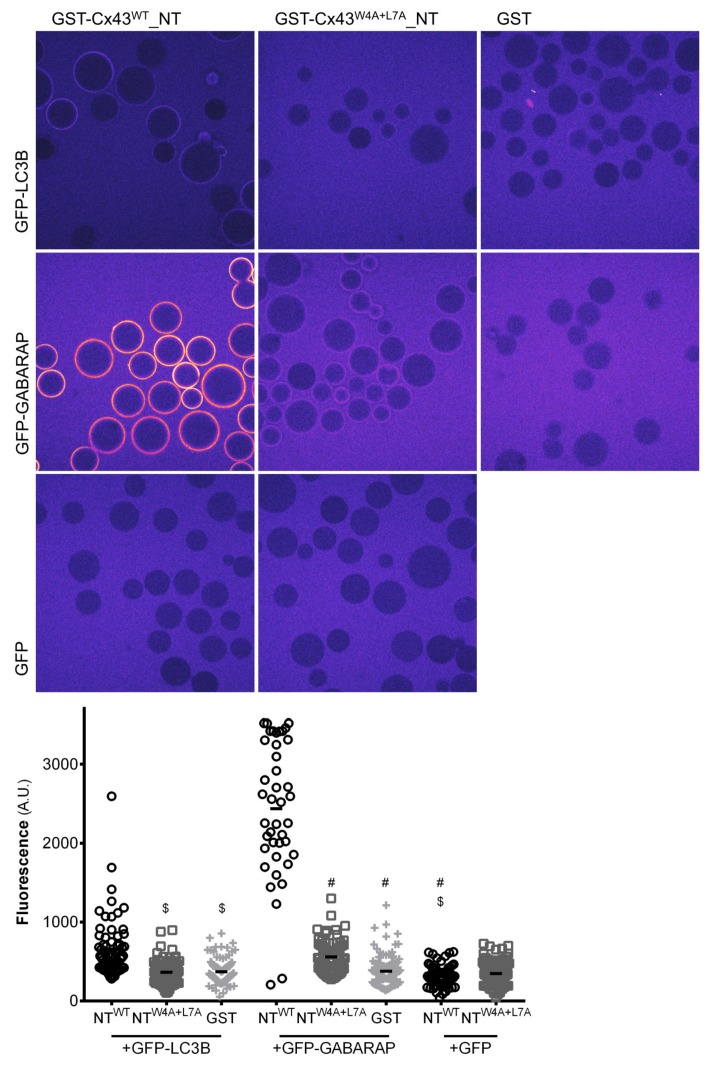
The LIR motif in the amino terminal of Cx43 interacts with GABARAP in vitro. Peptides comprising either the amino terminal of wild type (NT^WT^) or W4A+L7A mutant (NT^W4A+L7A^) Cx43 fused to GST were bound to glutathione beads and subsequently incubated with recombinant GFP-LC3B or GFP-GABARAP, after which the beads were transferred to a glass-bottom microplate and imaged using a confocal microscope. The GFP signal is shown in false colour (ImageJ: Fire). The intensity of the GFP signal surrounding the glutathione beads was measured and plotted in a graph. $ *p* < 0.0001 vs. NT^WT^+GFP-LC3B, # *p* < 0.0001 vs. NT^WT^+GFP-GABARAP using a Kruskal‒Wallis test, followed by Dunn’s multiple comparison test. Data represents four individual experiments.

To further ascertain that the LIR motif present in connexins can interact directly with LC3 proteins and is thus a bona fide functional motif we proceeded to conduct in vitro experiments using recombinant glutathione S-transferase (GST) proteins fused to the amino terminal of mutant or wild type Cx43. Cx43 was chosen as the model for these assays due to being the most widely expressed connexin in the body and the main focus in connexin research. Given that Cx43 is an integral transmembrane protein, it is difficult to produce and isolate the full-length protein from bacterial or insect protein production systems, and thus only the amino terminal was used in these pull-down experiments. As a first approach, we carried out pull-down experiments in which recombinant GFP-LC3B and GFP-GABARAP were bound to GFP-TRAP beads to serve as bait. GST-Cx43^WT^_NT and GST-Cx43^W4A+L7A^_NT were used as prey in the experiment. As depicted in Figure S2, there is a very clear interaction between GFP-GABARAP and GST-Cx43^WT^_NT. Importantly, when the LIR motif is mutated, this interaction is substantially decreased. Unexpectedly, we were not able to detect any interaction between GST-Cx43^WT^_NT and GFP-LC3B in this assay, even though the full-length form of Cx43 readily interacted with GFP-LC3B when co-expressed in HEK293A cells (Figure 1A,D). To further confirm this in vitro data, we performed a microscopy based protein-protein interaction assay [16], in which the GST-tagged Cx43^WT^_NT or Cx43^W4A+L7A^_NT constructs are first bound to glutathione beads and subsequently incubated with recombinant GFP-LC3 or GFP-GABARAP. The intensity of fluorescence formed around the glutathione beads reflects the interaction between Cx43 and either LC3B or GABARAP. Representative images displayed in Figure 2 show a strong interaction between GST-Cx43^WT^_NT and GFP-GABARAP, which is almost entirely abolished in the LIR mutant (middle row panels). We were also able to detect a weak interaction between GST-Cx43^WT^_NT and GFP-LC3B, which is also abolished when the LIR mutant is used (top row panels). No interaction was observed when Cx43 coated beads were incubated with GFP alone.

### 3.3. Cx43 and LC3B Interaction Occurs in Endocytic Compartments

**Figure 3:**
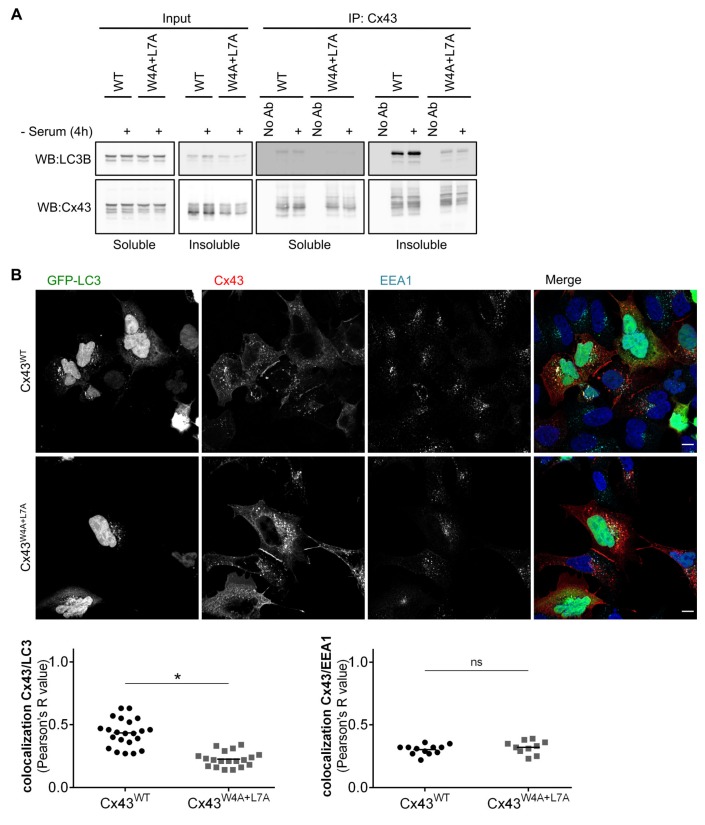
LC3B preferentially interacts with Cx43 present in post-endocytic vesicles. (**A**) HEK293A cells co-transfected with GFP-LC3B and wild type (WT) or mutant (W4A+L7A) Cx43 were incubated in medium without serum for 4 h (-Serum). Cell lysates were then separated into Triton X-100 soluble and insoluble fractions and immunoprecipitated with goat polyclonal antibodies directed against Cx43. Precipitates were then analysed using Western blot using rabbit polyclonal antibodies against Cx43 or LC3B. (**B**) HEK293A cells grown on coverslips were co-transfected with plasmids expressing GFP-LC3B and either Cx43^WT^ or Cx43^W4A+L7A^. Subcellular distribution of the proteins was evaluated using confocal microscopy, and co-localization of Cx43 (red) with GFP-LC3B (green) or EEA1 (cyan) was quantified and plotted in a graph. Scale bars, 10 μm. Dots represent values obtained for individual images (n = 4). *p < 0.05 using the Mann‒Whitney test.

Having established that the LIR motif present in Cx43 is functional, we sought to investigate where in the cell this interaction preferentially occurs. Triton X-100 fractionation assays are a crude method to separate free connexin hemichannels, which are predominantly recovered in the Triton X-100 soluble fraction, from GJ channels localized at plaques and internalized vesicles, which partition into the insoluble fraction [17]. Results depicted in Figure 3A show a clear preference for LC3B to interact with Cx43 present in the insoluble fraction. Serum starvation also appeared to induce an increase in the interaction in the insoluble fraction. Given that GJ plaques are internalized as a double membrane structure, these results suggest that LC3B interaction with Cx43 could occur at the plasma membrane or during the endocytosis/lysosomal pathway. To clarify this we evaluated the subcellular localization of Cx43 and GFP-LC3B through confocal immunofluorescence imaging. While the presence of Cx43WT and GFP-LC3B in the same intracellular compartments is easily apparent (Figure 3B, top panels), colocalization between Cx43^W4A+L7A^ and GFP-LC3B is much less discernible (Figure 3B, bottom panels), which was confirmed by quantification of the colocalized fluorescence signals. Taken together, these results suggest that Cx43 interaction with LC3B occurs mainly in internalized vesicles.

Mutation of the LIR motif of Cx43 appeared to not affect the stability of Cx43 at the plasma membrane, as demonstrated by its accumulation in GJ plaques at the cell surface. However, given the lower colocalization of Cx43^W4A+L7A^ with LC3-positive vesicles, when compared to Cx43^WT^, we sought to elucidate whether this mutation impairs Cx43 endocytosis. To address this question we performed confocal microscopy experiments using antibodies directed against early endosome antigen 1 (EEA1), a marker of early endosomes. Data presented in Figure 3B shows no significant difference between Cx43^WT^ and Cx43^W4A+L7A^ colocalization with EEA1, suggesting that mutation of the LIR motif of Cx43 does not affect its sorting into early endosomes and presumably its internalization. 

### 3.4. Tyrosine Residue 265 of Cx43 is Important for the Interaction with LC3B

**Figure 4:**
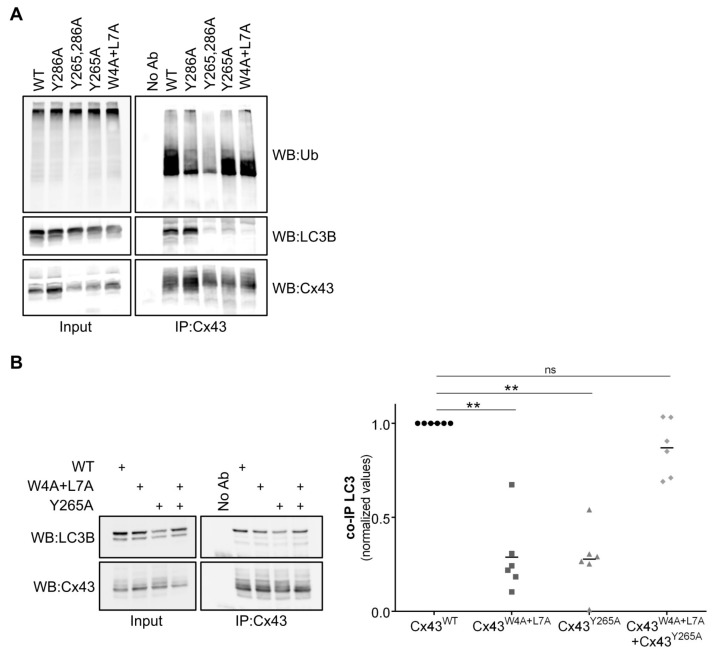
Tyrosine 265 of Cx43 is important for the interaction with LC3B. (**A**) HEK293A cells were co-transfected with GFP-LC3B and either wild type (WT) or mutant Cx43 (Y286A, Y265,286A, Y265A, W4A+L7A). Cell lysates were then subjected to immunoprecipitation with goat polyclonal antibodies against Cx43, and the precipitates were analysed using Western blot using mouse monoclonal antibodies against ubiquitin, or rabbit polyclonal antibodies against Cx43 and LC3B. (**B**) HEK293A cells co-transfected with GFP-LC3B and several combinations of wild type (WT) or mutant Cx43 (Y265A, W4A+L7A). Cell lysates were then subjected to immunoprecipitation with goat polyclonal antibodies against Cx43, and the precipitates were analysed using Western blot using rabbit polyclonal antibodies against Cx43 and LC3B. The level of co-immunoprecipitated LC3B with each connexin was quantified, normalized to the immunoprecipitated connexin and plotted in a graph. ** *p* < 0.01, ns: non-significant using a Kruskal‒Wallis test, followed by Dunn’s multiple comparison test.

In an attempt to narrow the possible cellular locations where the binding between Cx43 and LC3B occurs, we mutated tyrosine residues Y265 and Y286 on Cx43, which are part of two independent endocytic tyrosine-sorting signals, leading to the accumulation of Cx43 at the plasma membrane [20]. Tyrosine residue Y286 is also part of a proline-rich motif, which is required for Nedd4 binding and Cx43 ubiquitination (Table S1) [5,6,21]. Results presented in Figure 4A show that the ubiquitination defective mutant Cx43^Y286A^, with an intact LIR motif, displayed an intermediate behaviour between Cx43^WT^ and Cx43^W4A+L7A^ concerning the interaction with LC3B, reflecting the importance of ubiquitin in mediating the interaction between LC3 and Cx43. On the other hand, mutants Cx43^Y265,286A^ and Cx43^Y265A^ present a strong decrease in the interaction with LC3B. Considering that mutation of the Y265 residue does not significantly affect ubiquitination of Cx43^Y265A^ and the amino acids surrounding Y265 on Cx43 do not resemble the core consensus sequence for a LIR motif, structural changes likely underlie this effect.

Before being delivered to the plasma membrane, connexin proteins may oligomerize into hexameric structures containing different connexins called heteromeric hemichannels. Taking this into account, we evaluated the effect of co-expressing connexin mutants Cx43^W4A+L7A^ and Cx43^Y265A^ in the binding with LC3B. After establishing that these connexin mutants co-oligomerized into the same hemichannels (data not shown), we then co-expressed GFP-LC3B with both Cx43^W4A+L7A^ and Cx43^Y286A^ in HEK293A cells, and immunoprecipitated Cx43 from the lysates. Results depicted in Figure 4B show that when the two connexin mutants are co-expressed the binding to LC3B is restored, presumably due to the formation of heteromeric channels in which the non-mutated region of one protein can somehow compensate for the mutated region of the other. Thus, both the amino and carboxyl terminals of Cx43 play a role in LC3 binding.

### 3.5. LC3 Lipidation Is Not A Pre-Requisite for Binding to Cx43

LC3 family proteins exist in the cell as a non-lipidated form (LC3-I) or as a lipidated form when they are conjugated to PE (LC3-II). LC3-II is present on the membrane of phagophores where it can act as a binding location for LIR-motif containing autophagic receptors such as p62 [12]. Although the GFP-LC3B construct is indeed capable of being conjugated to PE, distinguishing its LC3-I and LC3-II forms on a Western blot is not always possible. Therefore, we sought to investigate if Cx43 binds preferentially to one of these forms through the use of HEK293A cells knocked down for ATG7, to impede the conjugation of LC3-I to PE. As observed in Figure S3, cells with lower levels of ATG7 present a defect in the lipidation of both GFP-LC3B and endogenous LC3B, as represented by the decrease in the lower molecular weight band corresponding to LC3B-II, even when autophagic degradation is interrupted by treatment with Bafilomycin A1 (Baf A1), which inhibits the fusion of autophagosomes with the lysosome. However, ATG7 depletion has no significant impact on the binding of GFP-LC3B to Cx43, suggesting that Cx43 interacts preferentially with LC3B-I.

### 3.6. Role of Phosphorylation in the Binding of Cx43 to LC3B

**Figure 5:**
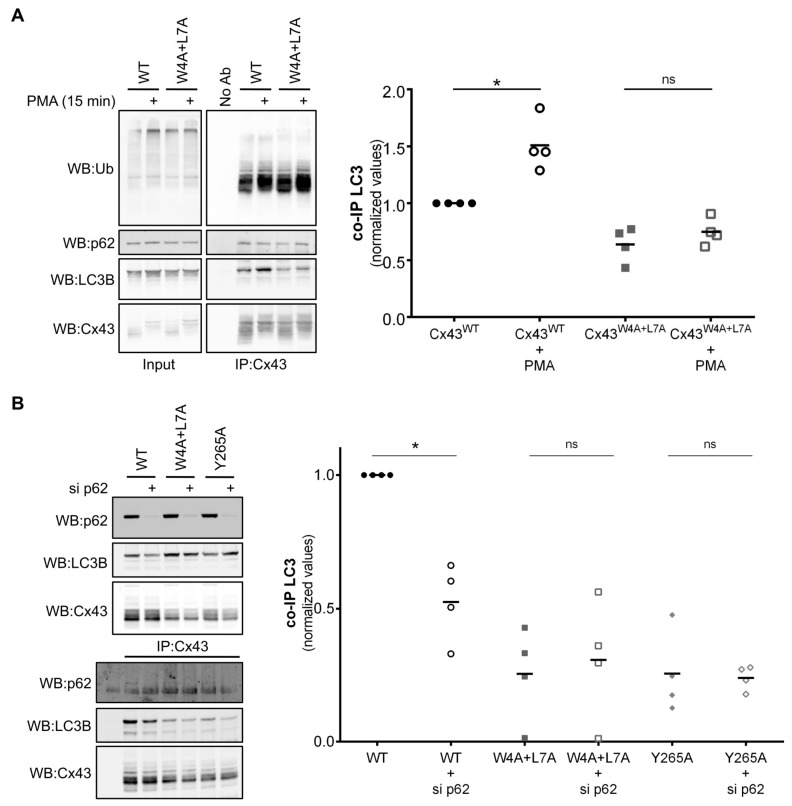
Role of phosphorylation and p62 in the binding of Cx43 to LC3B. (**A**) HEK293A cells co-transfected with GFP-LC3B and either wild type (WT) or mutant Cx43 (W4A+L7A) were incubated with 100 ng/mL PMA for 15 min. Cell lysates were then subjected to immunoprecipitation with goat polyclonal antibodies against Cx43, and the precipitates were analysed using Western blot using mouse monoclonal antibodies against ubiquitin, or rabbit polyclonal antibodies against Cx43, Nedd4 and LC3B. The level of co-immunoprecipitated LC3B with connexin in each condition was quantified, normalized to the immunoprecipitated connexin and plotted in a graph. *p < 0.05, ns: non-significant using the Mann‒Whitney test. (**B**) HEK293A cells were co-transfected with GFP-LC3B, either wild type (WT) or mutant Cx43 (W4A+L7A), and with siRNA directed against p62. Cells were harvested after 24 h, the cell lysates were then subjected to immunoprecipitation with goat polyclonal antibodies against Cx43 and the precipitates were analysed using Western blot using mouse monoclonal antibodies against p62, or rabbit polyclonal antibodies against Cx43 and LC3B. The level of co-immunoprecipitated LC3B with connexin in each condition was quantified, normalized to the immunoprecipitated connexin and plotted in a graph. *p < 0.05, ns: non-significant using the Mann‒Whitney test.

It has been established that Cx43 phosphorylation induces the ubiquitination of the protein and promotes its interaction with p62 [4,11]. Given that LC3B can interact directly with the LIR motif present on Cx43, we assessed the effect of Cx43 phosphorylation on this interaction. HEK293A cells co-transfected with GFP-LC3B and either wild type or Cx43 mutated in the LIR motif were treated with phorbol 12-myristate 13-acetate (PMA) to promote Cx43 phosphorylation and ubiquitination. As demonstrated in Figure 5A, PMA treatment leads to the accumulation of higher molecular weight forms of Cx43, consistent with Cx43 phosphorylation [22]. Furthermore, although PMA treatment induced an increase in the interaction of Cx43^WT^ with LC3B, this effect was milder for Cx43^W4A+L7A^, and insufficient to reach the interaction levels of the wild type protein. Notably, interaction of Cx43^W4A+L7A^ with p62 was only slightly decreased, indicating that binding to p62 does not rely on this LIR motif. Thus, although Cx43 phosphorylation does indeed promote the interaction between Cx43 and LC3 (in accordance with previous reports), it is insufficient to compensate for the absence of the LIR motif on Cx43.

### 3.7. Role of p62 in the Binding of Cx43 to LC3B

It has been previously shown that ubiquitination of Cx43 leads to the recruitment of the macroautophagy adaptor p62 and subsequent degradation of the protein [11]. As p62 can recruit cargo to nascent phagophores through its LIR-mediated binding to LC3, we evaluated the impact of depleting p62 in Cx43 binding to LC3B. Results presented in Figure 5B show that p62 depletion leads to a decrease in the interaction between LC3B and Cx43^WT^, whereas the already low interaction with the Cx43 mutants was not significantly altered. This suggests that although Cx43 interaction with LC3B is partially mediated by ubiquitination and p62 binding, this alone cannot overcome the loss of the LIR motif on Cx43.

### 3.8. GABARAP Interacts with the LIR Motif of Connexins

**Figure 6:**
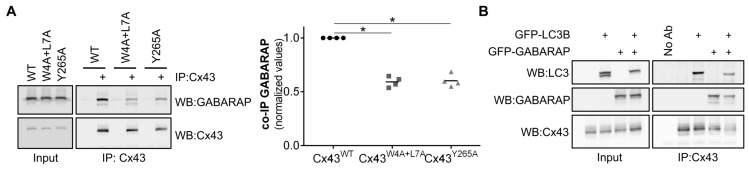
Mutation of the LIR motif of Cx43 impairs its binding to GABARAP and confers resistance to nutrient deprivation induced autophagy. (**A**) HEK293A cells were co-transfected with GFP-GABARAP and either wild type (WT) or mutant Cx43 (W4A+L7A, Y286A). Cell lysates were then subjected to immunoprecipitation with goat polyclonal antibodies against Cx43, and the precipitates were analysed using Western blot using rabbit polyclonal antibodies against Cx43 and GABARAP. The level of co-immunoprecipitated GABARAP with each connexin was quantified, normalized to the immunoprecipitated connexin and plotted in a graph. *p < 0.05, ns: non-significant using the Mann‒Whitney test. (**B**) HEK293A cells were co-transfected with Cx43 and either GFP-LC3B, GFP-GABARAP or both. Cell lysates were then subjected to immunoprecipitation with goat polyclonal antibodies against Cx43, and the precipitates were analysed using Western blot using rabbit polyclonal antibodies against Cx43, LC3B and GABARAP.

In vitro assays showed that GABARAP had a much stronger interaction with the LIR motif of Cx43 than LC3B (Figure 2 and Figure S2). To investigate this interaction in vivo, HEK293A cells were co-transfected with GFP-GABARAP and either wild type or mutant connexin. In accordance with the results obtained with LC3B, Cx43 binds to GABARAP and this interaction is reduced upon mutation of the LIR motif, and also when residue Tyrosine 265 is mutated to alanine (Figure 6A). Given that we observed a much stronger interaction between Cx43 and GABARAP in the in vitro assays, we investigated if co-expression of both LC3B and GABARAP could exert a dominant negative effect over the other for binding to Cx43. As observed in Figure 6B, when GFP-LC3B and GFP-GABARAP are co-expressed in HEK293A cells, neither protein has a special predominance for binding with Cx43.

We also investigated the binding of GABARAP to other connexins by co-transfecting HEK293A cells with GFP-GABARAP and either wild type or mutant Cx37, Cx40, Cx46 and Cx50 (Figure S4). In accordance with what was described above for LC3B, all connexins tested interacted with GABARAP. Likewise, mutation of the LIR motif inhibited this interaction for all connexins, with the exception of Cx40. Thus, the conserved LIR motif in the amino terminal of the connexin family appears to mediate the direct interaction with members of the LC3/GABARAP family.

### 3.9. Mutation of the LIR Motif of Cx43 Renders the Protein Resistant to Nutrient Deprivation Induced Autophagic Degradation

**Figure 7:**
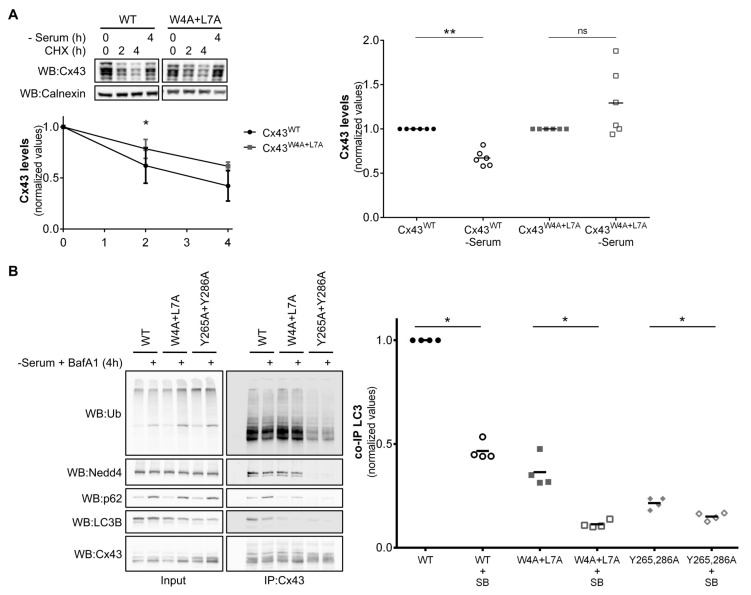
Mutation of the LIR motif of Cx43 renders the protein resistant to nutrient deprivation induced autophagic degradation. (**A**) HEK293A cells transfected with either wild type (WT) or mutant Cx43 (W4A+L7A) were either incubated with 50 µg/mL cycloheximide (CHX) for 2 or 4 h or subjected to starvation with media without serum for 4 h. Lysates were analysed using Western blot using goat polyclonal antibodies against Cx43 and calnexin. Cx43 levels were quantified, normalized to calnexin and plotted in a graph. *p < 0.05 using the multiple Student’s t-test with Holm‒Sidak’s correction. (**B**) HEK293A cells co-transfected with GFP-LC3B and either wild type (WT) or mutant Cx43 (W4A+L7A, Y265,286A) were incubated in media without serum supplemented with 50 µM BafA1 for 4 h (SB). Cell lysates were then subjected to immunoprecipitation with goat polyclonal antibodies against Cx43, and the precipitates were analysed using Western blot using mouse monoclonal antibodies against ubiquitin and p62, or rabbit polyclonal antibodies against Cx43, Nedd4 and LC3B. The level of co-immunoprecipitated LC3B with connexin in each condition was quantified, normalized to the immunoprecipitated connexin and plotted in a graph. *p < 0.05 using the Mann‒Whitney test.

Previous work from our laboratory demonstrated that nutrient deprivation induced Cx43 ubiquitination, p62 and Eps15 recruitment and, ultimately, Cx43 autophagic degradation [11]. We next investigated the role of the LIR motif of Cx43 during nutrient deprivation induced autophagy degradation of Cx43. HEK293A cells transfected with plasmids encoding either Cx43^WT^ or Cx43^W4A+L7A^ were incubated with media without serum for 4 h or with the protein synthesis inhibitor cycloheximide (CHX) for 2 or 4 h. As displayed in Figure 7A, CHX assays show that mutation of the LIR motif of Cx43 has a moderate effect in prolonging the half-life of the protein in basal conditions. In contrast, mutation of the LIR motif of Cx43 rendered the protein strongly resistant to nutrient deprivation induced autophagic degradation, suggesting a role for the motif in directing Cx43 to degradation in stress conditions.

We further assessed the importance of the LIR motif in the binding to LC3B during nutrient deprivation induced autophagy activation. In an attempt to preserve the interaction between LC3B and Cx43, HEK293A cells were incubated with the autophagosome/lysosome fusion inhibitor BafA1. In accordance, interaction with p62 is increased when the autophagy pathway is blocked, even in the case of Cx43^W4A+L7A^, as expected, since ubiquitination is not affected. Likewise, the Cx43^Y265,286A^ mutant presents a reduced interaction with Nedd4 and impaired ubiquitination, leading to less interaction with p62 and LC3B (Figure 7B). Surprisingly, we observed a decrease in the interaction between LC3B and Cx43, both in the wild type and the mutants, in the presence of BafA1, suggesting that Cx43 binding to LC3B, either directly or through ubiquitin/p62, is a transient process that occurs and is likely disrupted before the final autophagosome/lysosome fusion step. Alternatively, given that p62 interaction with Cx43 in the presence of BafA1 increases with the concomitant decrease in LC3B interaction, it is possible that binding of p62 to Cx43 is displacing LC3B from Cx43.

## 4. Discussion

Previous studies demonstrated that connexin proteins are degraded by autophagy. We and others have reported that degradation of Cx43 by macroautophagy requires prior ubiquitination of Cx43 and its further recognition by p62 [10,11]. However, preventing Cx43 ubiquitination by siRNA depletion of the E3 ubiquitin ligase Nedd4, or mutation of Tyrosine 286, a core residue in the PPxY motif used for Nedd4 binding to Cx43, failed to completely abrogate degradation by autophagy, suggesting that a parallel pathway, independent of Cx43 ubiquitination, may exist to deliver the protein to the autophagosome. In this manuscript we describe a LIR motif, present in the amino terminal, that is conserved in almost all human and rat connexin family members. Mutation of the core consensus residues tryptophan 4 and leucine 7 to alanine in Cx37, Cx43, Cx46 and Cx50 lead to a substantial decrease in binding to both LC3B and GABARAP. Moreover, in the case of Cx43, interaction with LC3B occurs mostly in internalized vesicles, as evidenced by the Triton X-100 fractionation experiments and immunofluorescence imaging. Lastly, mutation of the LIR motif of Cx43 also renders the protein resistant to nutrient deprivation induced autophagy. Data from each mutant is summarized in Table S1.

The results of in vitro protein-protein interaction assays suggest that the LIR motif present on the amino terminal of Cx43 binds preferentially to GABARAP as opposed to LC3B. A subset of LIR motifs that bind preferentially to GABARAP (GIM motif), characterized by the consensus sequence [W/F]-[V/I]-X2-V, was recently described [14]. Curiously, none of the LIR motifs present in the human and rat connexin family members can be considered as GIMs. More intriguing is the observation that this preference for binding to GABARAP present in the in vitro assays performed using only the amino terminal of Cx43 is not carried over to the interaction experiments using full-length Cx43 expressed in mammalian cells. Indeed, when GFP-LC3B is co-expressed with GFP-GABARAP, Cx43 does not display any binding preference. It is plausible that post-translational modifications, which are not available during the production of the GST-Cx43^WT^_NT chimera in bacteria, are necessary to facilitate the binding of Cx43 to LC3B. For instance, although the LIR consensus sequence is commonly defined as [W/F/Y]-X1-X2-[L/I/V], the presence of negative residues surrounding the core residues of the LIR motif has been described to modulate the interaction with LC3B [13]. Accordingly, it is conceivable that in vivo phosphorylation of amino acid residues surrounding the LIR motif of Cx43 promote interaction with LC3. Furthermore, changes in the topology and conformation adopted by Cxs when expressed in cells may justify the discrepancy of in vitro and in vivo data. Along the synthetic pathway, Cx monomers oligomerize to form a hexameric structure, the connexon, before reaching the plasma membrane. Thus, a fully mature Cx channel exists as a structure that would contain 6 LIR motifs in close proximity. Binding to LC3B in vivo may be facilitated by the presence of several LIR motifs clustered in a limited region and in a specific orientation, which is not easily replicated on the surface of the beads used in the in vitro experiments. Other regions of the protein may also facilitate the binding of LC3 to Cx43, as will be discussed further below.

Although all the five alpha subfamily connexins (Cx37, Cx40, Cx43, Cx46 and Cx50) tested can bind to LC3 proteins, only mutation of the LIR motif of Cx40 did not impair its interaction with either LC3B or GABARAP. It is possible that in the case of Cx40, the interaction we observed with LC3B/GABARAP occurred through other regions of the protein or was mediated by ubiquitination. Moreover, differences in the amino terminal of Cx40 may also contribute to render the LIR motif nonfunctional. It will be interesting to investigate this issue in the future considering that Cx40 can co-oligomerize and is found co-expressed with Cx37 and Cx43 in several tissues.

Surprisingly, we demonstrated that mutation of Tyrosine 265 on the carboxyl terminal of Cx43 also prevented binding to LC3B and GABARAP, although this residue was not part of any discernible LIR motif. Tyrosine 265 and 286 are part of two different endocytic tyrosine-sorting signals that were previously shown to play a role in the endocytosis of Cx43 [6,20], whereas Tyrosine 286 is additionally part of a PPxY motif required for binding to Nedd4 and subsequent Cx43 ubiquitination [5,21]. At a first glance, the impaired interaction of Cx43^Y265A^ with LC3B may suggest that the interaction could only occur after Cx43 internalization. Nevertheless, Cx43^Y286A^, which also accumulates at the plasma membrane, still retains a relatively high binding level to LC3B, although to a lesser extent when compared to Cx43^WT^, likely due to ubiquitination defects that hamper p62-mediated binding. Thus, some other mechanism, beyond the mere subcellular localization of Cx43^Y265A^ must be in play to explain its diminished binding to LC3B. Curiously, when Cx43^W4A+L7A^ was co-expressed with Cx43^Y265A^, binding to LC3B was restored to normal levels. Presumably, when the two mutants oligomerize into the same hemichannel, they are able to compensate for each other’s mutation. This suggests a model in which direct LC3B binding to Cx43 depends not only on the LIR motif in the amino terminal, but also on the carboxyl terminal of another Cx present in the hemichannel. Furthermore, it is plausible that more than a strict dependence on Y265, mutation of this residue simply induces a conformational change in the carboxyl terminal that physically prevents access of LC3B to the LIR motif. It has been reported that Y265 phosphorylation by Src kinase impacts on different aspects of the Cx43 life cycle [23], raising the possibility that Y265 phosphorylation may also modulate binding to LC3B. Another intriguing possibility is that the interaction of LC3B with Cx43 requires two binding sites, the LIR motif on the amino terminal, which would bind to the LIR docking site on LC3B, and an additional region in the carboxyl terminal, either including or modulated by Y265. Notably, LC3B does not bind to other proteins exclusively through its LIR docking site [12]. Indeed, it has recently been reported that LC3B can interact with ubiquitin interacting motifs (UIM) through an UIM-docking site (UDS) [24], and also with cardiolipin [25] and Lamin B1 [26].

Triton X-100 fractionation experiments showed that the interaction occurred mainly in the Triton X-100 resistant fraction, which contains Cx43 present in GJ plaques and also internalized GJs. In addition, immunofluorescence imaging showed that the two proteins colocalized mainly in intracellular vesicles, thus supporting a model in which Cx43/LC3B interaction occurs mainly in internalized compartments. Treatment with PMA, which induces Cx43 phosphorylation, ubiquitination and internalization, lead to an increase in LC3B binding to Cx43^WT^, while having no effect on binding with Cx43^W4A+L7A^, suggesting that inducing the phosphorylation and ubiquitination of Cx43 cannot overcome the lack of a functional LIR motif. However, and in accordance with previous reports [11], Cx43 ubiquitination still plays an important role in LC3B binding, as shown by the decrease in interaction when p62 is depleted. Our ATG7 silencing experiments also suggest that Cx43 binds preferentially to the non-conjugated form of LC3B, as while ATG7 depletion decreased the levels of LC3B-II, binding of LC3B to Cx43 was not impaired. This data raises an important question as to the function of this direct interaction of Cx43 with LC3B, within the context of the existence of a parallel pathway involving ubiquitination and p62 binding. An important fact to consider is that an autophagy substrate containing multiple points of LC3 interaction is not without precedent. For example, during mitophagy, ubiquitinated proteins on the surface of the mitochondria recruit autophagy receptors to assist in targeting the mitochondria to autophagosomes [12]. However, in addition to this mechanism, LIR motif containing proteins on the surface of the mitochondria, such as Nix and FUNDC1, can also bind directly to LC3 to assist in mitophagy [27]. In addition, the phospholipid cardiolipin, which is transported to the outer mitochondrial membrane following damage, can also bind to LC3 in a non-LIR motif dependent manner [25]. Although plasma membrane proteins committed for degradation usually reach the lysosome through the endolysosomal pathway, GJs can be delivered to lysosomes through autophagy. This particularity of Cx proteins may stem from their unique internalization mechanism. Plasma membrane proteins are normally internalized through single membrane structures, whereas GJ plaques are internalized into one of the adjacent cells through a double membrane structure commonly referred to as an annular gap junction (AGJ). Perhaps the structure of AGJs is unsuited for easy processing through the endolysosomal pathway, requiring their degradation through autophagy. In such a case, as with mitophagy, the presence of multiple binding spots for LC3 may allow for more efficient engulfment of the AGJ by the phagophore. Another intriguing possibility is that this interaction is used by Cx43 to promote the recruitment of non-conjugated LC3B to AGJs to assist with its degradation by macroautophagy. Notably, Cx43 is also known to interact with several autophagy-related proteins, including ATG16, Vps35, Beclin-1 and Vps15 [28], raising the possibility that Cx43 may function as a scaffold for autophagy machinery to further facilitate phagophore formation around AGJs. Although LC3/GABARAP family members offer some redundancy in function, specific functions for particular family members have been described, with the LC3 subfamily participating in the elongation of the phagophore membrane, while the GABARAP subfamily is involved in later stages of autophagosome formation [12]. The ability of Cxs to interact with both subfamilies envisions a model in which the sequential binding of Cx43 to different LC3/GABARAP subfamily members is important to ensure AGJ engulfment and processing during autophagosome maturation. 

Altogether, the results presented in this work lead us to propose a model in which Cxs bind directly to LC3/GABARAP proteins to facilitate their degradation by autophagy. Given the importance of GJIC in multiple tissues and organs, and its involvement in specific pathologies such as heart failure, understanding the mechanisms that regulate connexin degradation may shed new light upon the role of the connexin family in these diseases.

## Supplementary Materials

The following are available online at https://www.mdpi.com/2073-4409/9/4/902/s1, Figure S1: Comparison of the amino acid sequences of the amino terminal domains of human and rat connexins, Figure S2: The LIR motif in the amino terminal of Cx43 interacts with GABARAP in vitro, Figure S3: LC3B lipidation is not required for the interaction with Cx43, Figure S4: Mutation of the LIR motif of Cx37, 46 and 50 impairs their interaction with GABARAP, Table S1: Description of connexin mutants and their effect on binding to LC3/GABARAP proteins, Graphical Abstract.
