# Supplementary material for: A Conserved LIR Motif in Connexins Mediates Ubiquitin-Independent Binding to LC3/GABARAP Proteins
Source: Cells. 2020 Apr 7;9(4):902. doi: 10.3390/cells9040902 (PMC7226732; doi:10.3390/cells9040902)
Supplement: Supplementary file 1 [file cells-09-00902-s001.zip › Figures S1 & S2.pdf]

A

GJA4	Cx37				M	G	D	W	G	F	L	E	K	L	L	D	Q		V	Q	E	H	S	T	V	V	G	K
GJA5	Cx40				M	G	D	W	S	F	L	G	N	F	L	E	E		V	H	K	H	S	T	V	V	G	K
GJA1	Cx43				M	G	D	W	S	A	L	G	K	L	L	D	K		V	Q	A	Y	S	T	A	G	G	K
GJA3	Cx46				M	G	D	W	S	F	L	G	R	L	L	E	N		A	Q	E	H	S	T	V	I	G	K
GJA8	Cx50				M	G	D	W	S	F	L	G	N	I	L	E	E		V	N	E	H	S	T	V	I	G	R
GJA9	Cx59				M	G	D	W	N	L	L	G	D	T	L	E	E		V	H	I	H	S	T	M	I	G	K
GJA10	Cx62				M	G	D	W	N	L	L	G	G	I	L	E	E		V	H	S	H	S	T	I	V	G	K
GJB7	Cx25				M		S	W	M	F	L	R	D	L	L	S	G		V	N	K	Y	S	T	G	T	G	W
GJB2	Cx26				M		D	W	G	T	L	Q	T	I	L	G	G		V	N	K	H	S	T	S	I	G	K
GJB6	Cx30				M		D	W	G	T	L	H	T	F	I	G	G		V	N	K	H	S	T	S	I	G	K
GJB4	Cx30.3				M		N	W	A	F	L	Q	G	L	L	S	G		V	N	K	Y	S	T	V	L	S	R
GJB3	Cx31				M		D	W	K	T	L	Q	A	L	L	S	G		V	N	K	Y	S	T	A	F	G	R
GJB5	Cx31.1				M		N	W	S	I	F	E	G	L	L	S	G		V	N	K	Y	S	T	A	F	G	R
GJB1	Cx32				M		N	W	T	G	L	Y	T	L	L	S	G		V	N	R	H	S	T	A	I	G	R
GJC3	Cx30.2/Cx31.3				M		C	G	R	F	L	R	R	L	L	A	E		E	S	R	R	S	T	P	V	G	R
GJC1	Cx45				M		S	W	S	F	L	T	R	L	L	E	E		I	H	N	H	S	T	F	V	G	K
GJC2	Cx47	M	T	N	M		S	W	S	F	L	T	R	L	L	E	E		I	H	N	H	S	T	F	V	G	K
GJD3	Cx31.9				M	G	E	W	A	F	L	G	S	L	L	D	A		V	Q	L	Q	S	P	L	V	G	R
GJD2	Cx36				M	G	E	W	T	I	L	E	R	L	L	E	A	A	V	Q	Q	H	S	T	M	I	G	R
GJD4	Cx40.1				M	E	G	V	D	L	L	G	F	L	I	I	T		L	N	C	N	V	T	M	V	G	K
Consensus					M	-	D	W	-	F	L	-	-	L	L	-	-		V	-	-	H	S	T	-	-	G	K

B

GJA6	Cx33				M	S	D	W	S	A	L	H	Q	L	L	E	K		V	Q	P	Y	S	T	A	G	G	K
GJA4	Cx37				M	G	D	W	G	F	L	E	K	L	L	D	Q		V	Q	E	H	S	T	V	V	G	K
GJA5	Cx40				M	G	D	W	S	F	L	G	E	F	L	E	E		V	H	K	H	S	T	V	I	G	K
GJA1	Cx43				M	G	D	W	S	A	L	G	K	L	L	D	K		V	Q	A	Y	S	T	A	G	G	K
GJA3	Cx46				M	G	D	W	S	F	L	G	R	L	L	E	N		A	Q	E	H	S	T	V	I	G	K
GJA8	Cx50				M	G	D	W	S	F	L	G	N	I	L	E	E		V	N	E	H	S	T	V	I	G	R
GJA10	Cx57				M	G	D	W	N	L	L	G	G	I	L	E	E		V	H	S	H	S	T	I	V	G	K
GJB2	Cx26				M		D	W	G	T	L	Q	S	I	L	G	G		V	N	K	H	S	T	S	I	G	K
GJB6	Cx30				M		D	W	G	T	L	H	T	V	I	G	G		V	N	K	H	S	T	S	I	G	K
GJB4	Cx30.3				M		N	W	G	F	L	Q	G	I	L	S	G		V	N	K	Y	S	T	A	L	G	R
GJB3	Cx31				M		D	W	K	K	L	Q	D	L	L	S	G		V	N	Q	Y	S	T	A	F	G	R
GJB5	Cx31.1				M		N	W	S	V	F	E	G	L	L	S	G		V	N	K	Y	S	T	A	F	G	R
GJB1	Cx32				M		N	W	T	G	L	Y	T	L	L	S	G		V	N	R	H	S	T	A	I	G	R
GJC1	Cx45				M		S	W	S	F	L	T	R	L	L	E	E		I	H	N	H	S	T	F	V	G	K
GJC2	Cx47	M	T	N	M		S	W	S	F	L	T	R	L	L	E	E		I	H	N	H	S	T	F	V	G	K
GJD2	Cx36				M	G	E	W	T	I	L	E	R	L	L	E	A	A	V	Q	Q	H	S	T	M	I	G	R
GJD4	Cx39				M	E	N	L	N	L	L	G	F	L	F	I	T		L	N	C	N	V	T	I	M	G	M
Consensus					M	-	D	W	-	-	L	-	-	L	L	-	-		V	N	-	H	S	T	-	-	G	K

Figure S1. Comparison of the amino acid sequences of the amino terminal domains of the 20 human (**A**) [1] and 17 rat (**B**) connexins. The connexins are grouped according to sub-families (GJA, GJB, etc.) and then ordered by increasing molecular weight. Gaps were allowed to maximize the identities among sequences. The consensus sequence requires ≥50% identity at a given position. The conserved LIR residues are marked in colour.

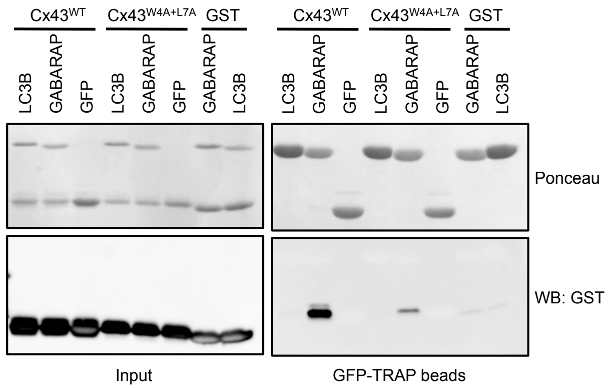

Figure S2. The LIR motif in the amino terminal of Cx43 interacts with GABARAP in vitro. **(A)** Recombinant GFP-LC3B or GFP-GABARAP were bound to GFP-Trap beads and subsequently incubated with peptides comprising the amino terminal of wild type (NT^{WT}) or W4A+L7A mutant ($NT^{W4A+L7A}$) Cx43 fused to GST. Precipitates were then analysed by Western Blot using goat polyclonal antibodies against GST.
