# Supplementary material for: A Conserved LIR Motif in Connexins Mediates Ubiquitin-Independent Binding to LC3/GABARAP Proteins
Source: Cells. 2020 Apr 7;9(4):902. doi: 10.3390/cells9040902 (PMC7226732; doi:10.3390/cells9040902)
Supplement: Supplementary file 1 [file cells-09-00902-s001.zip › Figures S3 & S4.pdf]

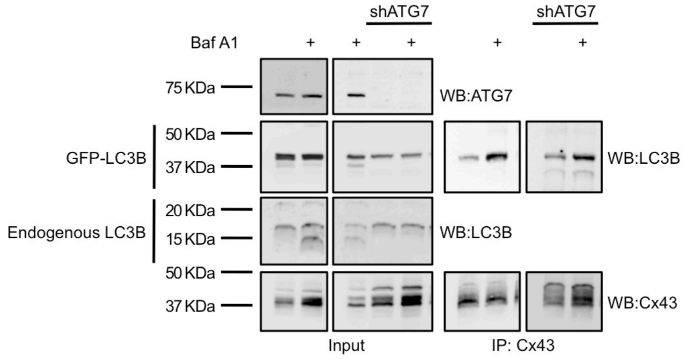

Figure S3. LC3B lipidation is not required for the interaction with Cx43. HEK293A cells were infected with a lentivirus encoding an shRNA directed against ATG7. After confirming ATG7 depletion, cells were co-transfected with GFP-LC3B and wild type Cx43 and incubated with 50μM BafA1 for four hours. Cell lysates were then subjected to immunoprecipitation with goat polyclonal antibodies against Cx43 and the precipitates were analysed by Western blot using rabbit polyclonal antibodies against Cx43 and LC3B.

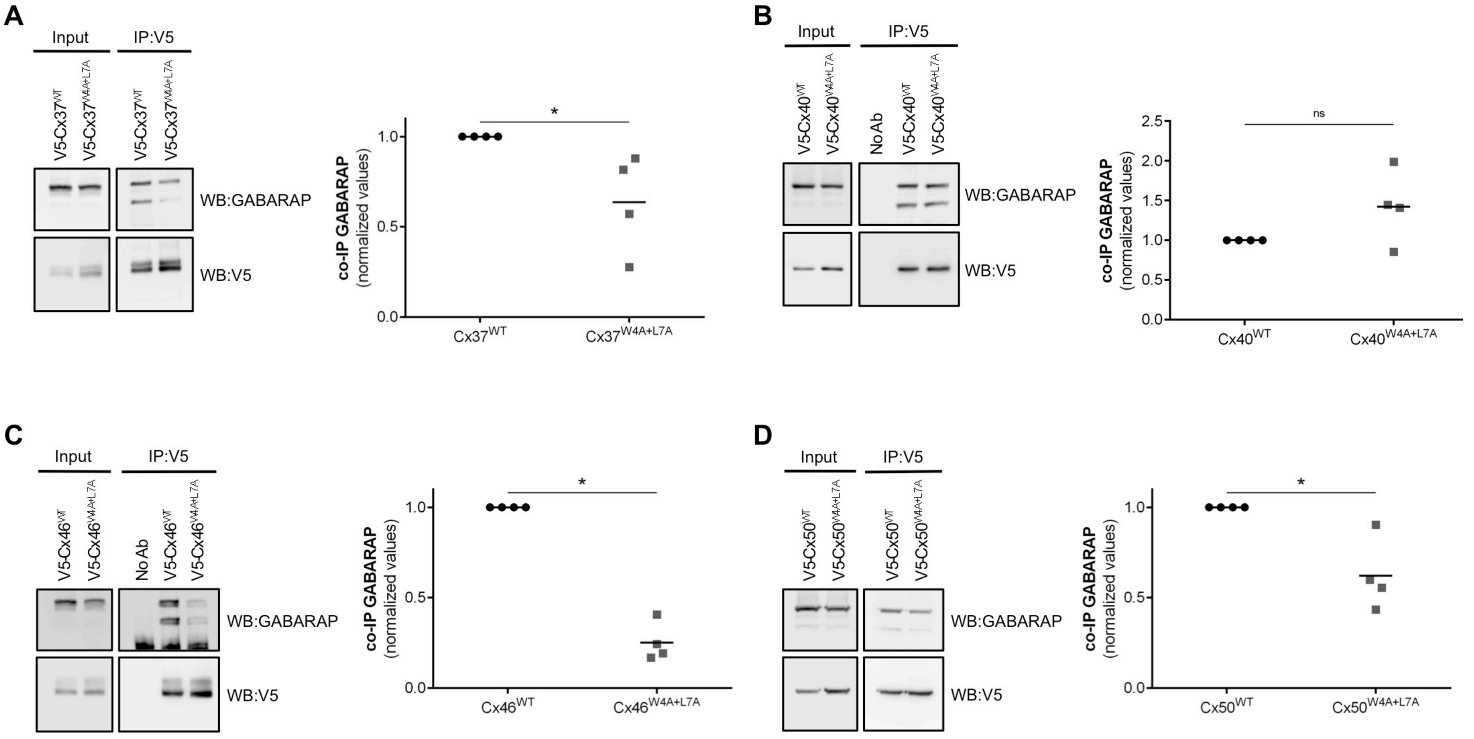

Figure S4. Mutation of the LIR motif of Cx37, 46 and 50 impairs their interaction with GABARAP. HEK293A cells were co-transfected with GFP-GABARAP and V5 constructs of either wild type or W4A+L7A mutants of Cx37 (**A**), Cx40 (**B**), Cx46 (**C**) and Cx50 (**D**). Cell lysates were then subjected to immunoprecipitation with goat polyclonal antibodies against V5. The precipitates were analysed by Western blot using mouse monoclonal antibodies against V5, or rabbit polyclonal antibodies against GABARAP. Level of co-immunoprecipitated GABARAP with each connexin was quantified, normalized to the immunoprecipitated connexin and plotted. *$p<0.05$, ns: non significant using Mann-Whitney test.
