# Supplementary material for: A Conserved LIR Motif in Connexins Mediates Ubiquitin-Independent Binding to LC3/GABARAP Proteins
Source: Cells. 2020 Apr 7;9(4):902. doi: 10.3390/cells9040902 (PMC7226732; doi:10.3390/cells9040902)
Supplement: Supplementary file 1 [file cells-09-00902-s001.zip › Table S1.pdf]

**Connexin protein**	**Details**	**Interaction with LC3/GABARAP**
Cx43$^{W4A+L7A}$	Mutation of the LIR motif	Decreased
Cx43^{Y265A}	Mutation of tyrosine-sorting signal<br>Accumulates on the plasma membrane	Decreased
Cx43^{Y286A}	Mutation of tyrosine-sorting signal and PPxY motif<br>Accumulates at the plasma membrane<br>Impaired ubiquitination	Moderately decreased
Cx43^{Y265,286A}	Mutation of both tyrosine-sorting signals and PPxY motif<br>Accumulates at the plasma membrane<br>Impaired ubiquitination	Decreased
GST-Cx43$^{W4A+L7A}$_NT	Mutation of the LIR motif<br>Amino-terminal of protein	Decreased
Cx37$^{W4A+L7A}$	Mutation of the LIR motif	Decreased
Cx40$^{W4A+L7A}$	Mutation of the LIR motif	Unaffected
Cx46$^{W4A+L7A}$	Mutation of the LIR motif	Decreased
Cx50$^{W4A+L7A}$	Mutation of the LIR motif	Decreased

Table S1. Description of connexin mutants and their effect on binding to LC3/GABARAP proteins.
